# Calculating Volume of Pig Point Cloud Based on Improved Poisson Reconstruction

**DOI:** 10.3390/ani14081210

**Published:** 2024-04-17

**Authors:** Junyong Lin, Hongyu Chen, Runkang Wu, Xueyin Wang, Xinchang Liu, He Wang, Zhenfang Wu, Gengyuan Cai, Ling Yin, Runheng Lin, Huan Zhang, Sumin Zhang

**Affiliations:** 1 College of Mathematics and Informatics, South China Agricultural University, Guangzhou 510642, China; 202125220611@stu.scau.edu.cn (J.L.); yu2_yu4@stu.scau.edu.cn (H.C.); 13242701096@163.com (R.W.); 15818982305@163.com (X.W.); starlxc0313@gmail.com (X.L.); wanghe20021212@163.com (H.W.); lrh870657823@gmail.com (R.L.); suminzhang@scau.edu.cn (S.Z.); 2 National Engineering Research Center for Swine Breeding Industry, Guangzhou 510642, China; cgy0415@163.com; 3 College of Animal Science, South China Agricultural University, Guangzhou 510642, China; 4 State Key Laboratory of Swine and Poultry Breeding Industry, Guangzhou 510640, China; 5 College of Foreign Studies, South China Agricultural University, Guangzhou 510642, China; 13640261933@163.com

**Keywords:** pig, volume calculation, point cloud reconstruction, weight estimation

## Abstract

**Simple Summary:**

The volume of a pig, a new phenotype feature, can be used to estimate its weight due to its high correlation with body weight. The proportions of different body parts, such as the head and legs, can be determined through point cloud segmentation, providing new phenotype information for breeding pigs with smaller heads and stouter legs. However, due to the irregular shape and potential missing parts of the pig point cloud, it is challenging to form a closed surface for volume calculation. The study addresses this challenge by using an improved Poisson reconstruction algorithm, which provides smoother, more continuous, and complete reconstruction results, and its accuracy and reliability have been confirmed. The study also found that the correlation coefficient between pig body volume and weight was 0.95, indicating a strong relationship. This research could be valuable for improving the efficiency and accuracy of livestock weight estimation and breeding.

**Abstract:**

Pig point cloud data can be used to digitally reconstruct surface features, calculate pig body volume and estimate pig body weight. Volume, as a pig novel phenotype feature, has the following functions: (a) It can be used to estimate livestock weight based on its high correlation with body weight. (b) The volume proportion of various body parts (such as head, legs, etc.) can be obtained through point cloud segmentation, and the new phenotype information can be utilized for breeding pigs with smaller head volumes and stouter legs. However, as the pig point cloud has an irregular shape and may be partially missing, it is difficult to form a closed loop surface for volume calculation. Considering the better water tightness of Poisson reconstruction, this article adopts an improved Poisson reconstruction algorithm to reconstruct pig body point clouds, making the reconstruction results smoother, more continuous, and more complete. In the present study, standard shape point clouds, a known-volume Stanford rabbit standard model, a measured volume piglet model, and 479 sets of pig point cloud data with known body weight were adopted to confirm the accuracy and reliability of the improved Poisson reconstruction and volume calculation algorithm. Among them, the relative error was 4% in the piglet model volume result. The average absolute error was 2.664 kg in the weight estimation obtained from pig volume by collecting pig point clouds, and the average relative error was 2.478%. Concurrently, it was determined that the correlation coefficient between pig body volume and pig body weight was 0.95.

## 1. Introduction

During the growth stage of pigs, body size measurement, weight estimation and other information can be harnessed to monitor pig growth, daily weight gain, and feed weight gain ratio and for science-based pig breeding [1]. Pig body weight and pig body size are the two most important evaluation indicators for phenotypic group selection and lineage performance. In particular, pig body weight is an important factor in managing the supply chain at various stages [2], and body weight information can be used to estimate pig growth, feed conversion efficiency, and disease occurrence rate [3].

Currently, weight estimation methods mainly adopt electronic scales for automatic measurement, which still requires strenuous manual labor. And the measurement accuracy of the electronic scales is easily affected by various factors such as the movement, urination, and defecation of pigs. Moreover, electronic scales are prone to erosion when placed in a pig farm, and the maintenance cost is relatively high. In order to solve the above problems, numerous researchers have conducted many studies on contactless body weight estimation of pigs. The common methods are to obtain the two-dimensional images of pigs and collect pig body size data or projected contour area. By establishing a correlation prediction model concerning body size, projected area, and body weight, pig weight estimation can be achieved [4,5,6,7]. Chen et al. [8] accurately predicted pig weight by constructing a deep neural network to automatically measure multiple body parameters. Nguyen et al. [9] collected RGB-D data of fattening pigs using a handheld camera. By computing the 3D point clouds of each target pig, latent features from a 3D generative model were employed to predict pig weight using three regression models (SVR, MLP, and AdaBoost). Thapar et al. [10] developed a novel, portable, and user-friendly method for hands-off body dimension measurement and body weight prediction of the Ghoongroo pig by using a smartphone with an object measurement app—On 3D CameraMeasure. Cang et al. [11] obtained depth images of the pig’s back from the top view camera and input them into a deep neural network to implement body weight estimation. Li et al. [5] acquired the key body size information extracted from the point clouds of a pig’s back and used it as independent variables for regression analysis models to estimate body weight. However, it is a tough task to collect relevant parameters on a curved surface of a pig body by using 2D cameras or single-view 3D cameras, and therefore, difficult to further improve the accuracy of pig body weight estimation in multi-scenario application. Further research has shown that three-dimensional parameters such as volume can more intuitively present animal phenotypes and are highly correlated with their body weight [12].

Unlike two-dimensional images, the volume information contains three-dimensional information of the curved surface of the pig body, which compensates for the insufficient dimensionality estimation of 2D image estimation methods and enhances the accuracy of body weight estimation. Some scholars at home and abroad have used volume as a major parameter for estimating livestock weight. The main approach consists of three steps: the first step is to obtain depth images of the pig’s back; the second step is to calculate the pig’s volume parameters by combining the back area; the final step is to establish a model using volume, body weight, and other parameters for the purpose of weight estimation [13,14,15,16]. Fu et al. [17] approximated the head and torso of a pig as a cone and a cylinder, respectively, thus obtaining a simplified three-dimensional model of a breeding pig. They then established a weight estimation model based on the volume of the three-dimensional model and weight to estimate the body weight of the breeding pig. Chu used the surface area obtained from point cloud reconstruction and volume parameters excluding limbs and head to construct a model to estimate the body weight of dairy cows, with high accuracy achieved in the estimation results [18]. Using a 3D point cloud is a non-contact weight measurement method to calculate the volume and the estimated body weight of a pig. Additionally, the volume of each pig body part can be obtained via point cloud segmentation [19]; therefore, more phenotypic features of pigs that cannot be acquired by manual measurement are available.

The traditional method for calculating the volume of complex objects is the drainage method: by completely immersing the object in water, the volume of the object can be calculated based on the difference between water surface heights before and after immersion in the area of the container. As pigs are living organisms, applying this method requires customized containers, which is difficult to implement in actual scenarios.

Thanks to the rapid development of consumer-grade 3D acquisition devices, low-cost and lightweight 3D acquisition devices such as Morpho3D, Kinect, and ASUS Xtion Pro have entered into the research field of intelligent agriculture, particularly in animal husbandry. Hence, the research on non-contact automatic measuring systems for body size of animals such as pigs, cows, and sheep has been widely carried out [5,14,20,21,22]. Currently, researchers use two or more depth cameras from different angles to collect surface point clouds of large-sized quadruped animals such as live pigs and cows and automatically measure various body sizes such as body length, body height, thoracic circumference, abdominal circumference, and chest depth by fusing complete point clouds [19,21,23,24,25,26,27,28,29]. There are also studies using deep learning models to position key body points on RGB images and using intrinsic camera parameters to project the detected key points onto the surface of livestock point clouds, thus achieving body size measurement of pigs and cows [30,31]. Up-to-date research has focused on automatic and accurate animal body measurement using three-dimensional surface point clouds [32]. Meanwhile, the three-dimensional phenotype reconstruction model has also brought new thoughts for calculating the volume, surface area, and volume of various parts of animals with the aid of point clouds.

At present, the research on the calculation method of irregular object point cloud volume is still in the growth stage, and there is much room to be desired for the study of calculation algorithm for complex object point cloud volume. The calculation methods related to irregular object point cloud model volume mainly include the slice method [33], Monte Carlo method, and model reconstruction method [34,35].

**Figure 1:**
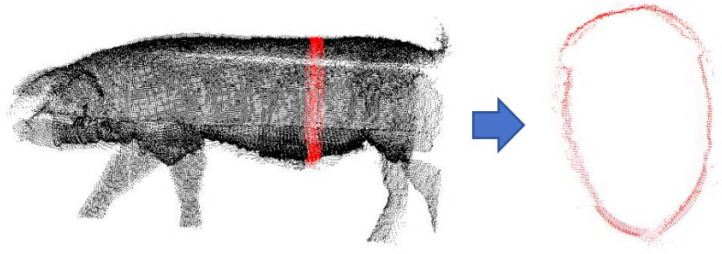
Slicing method for slice contour extraction.

In 2016, Zhi et al. [36] proposed a 3D point cloud volume calculation method based on the slicing method. As shown in Figure 1, this method mainly uses the idea of slice integration to partition point cloud data, determine the slice contour, calculate the area of each part, and multiply the area by the slice interval to obtain the approximate volume of the slices; thus, individual data can add up to the overall point cloud volume.

Subsequently, researchers improved the method of slicing, calculating volume from diverse aspects such as slice spacing, selection of inner and outer contour projections for slice projection, and cutting direction [37,38,39]. As the most classic three-dimensional volume calculation method, the slicing method has good accuracy and operation efficiency in calculating volume when handling simple objects such as crown canopy, coal stockpiles, and grain piles. However, as for complex objects, it is necessary to consider the errors caused by point cloud contour fitting (inner and outer), point cloud multi-contour fitting, as well as cutting direction and cutting interval. The missing point clouds in volume calculation will affect the water tightness of the 3D reconstruction model, so it is essential to obtain high-quality preprocessed point clouds for volume calculation.

Using the Monte Carlo method to calculate volume is a very representative algorithm [40,41,42] that involves the probability of random sampling points inside or outside the 3D model as the solution to the problem. This method is characterized by using random sampling to obtain approximate results. As the number of sampling times increases, the probability of obtaining correct results gradually increases. Conversely, if the sampling is not sufficient, it will cause obvious errors [43]. The Monte Carlo method requires that a large number of random points be inserted to improve the calculation accuracy. Judging whether the random points are inside the model or not is time-consuming [44]; therefore, Monte Carlo method is more frequently used to calculate the volume of smaller objects and it is suitable for closed models.

The model reconstruction method first reconstructs the point cloud into a mesh model structure, then decomposes the mesh model into multiple polyhedra, and finally sums the volumes of all polyhedra to obtain the total volume. The advantage of this method is that it converts discrete point cloud data into a surface model with continuity, creating a high-precision digital model that better preserves the geometric shape and surface features of the point cloud data. At the same time, it makes the calculated volume closer to the original volume.

In summary, for complex pig point clouds, solving the point cloud volume based on the model reconstruction method is an ideal option. However, preprocessing of point cloud data is required prior to model reconstruction to lessen the impacts of point cloud data quality, noise, and voids on the reconstruction results. Meanwhile, selecting appropriate model reconstruction algorithms and parameters to adapt to the characteristics of pig point clouds is also a crucial step.

Mesh model reconstruction algorithms primarily include the Delaunay triangulation method [45,46] and Poisson’s reconstruction method [47,48]. Delaunay performs non-overlapping triangulation on point clouds to generate a mesh model composed of triangles, while Poisson reconstruction interpolates and fits point cloud data using Poisson’s equations to generate a smooth curved surface model. For comparison, Delaunay is sensitive to the noise and voids present in point clouds, which may lead to incomplete and inaccurate reconstruction results. Poisson reconstruction has a certain degree of robustness and can fill the voids and smooth the surface to a certain extent.

**Figure 2:**
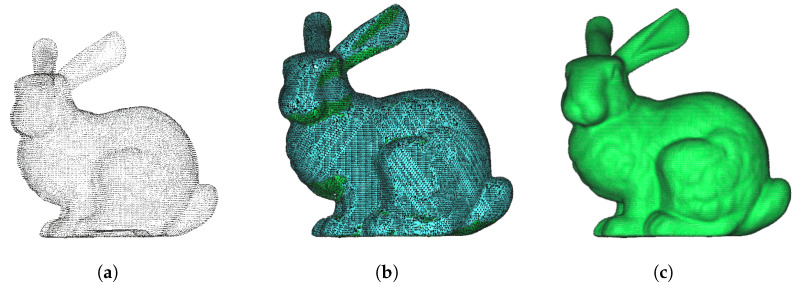
Comparison of Stanford rabbit [49] point cloud reconstruction. (**a**) Stanford rabbit point cloud. (**b**) Delaunay triangulation algorithm. (**c**) Poisson reconstruction algorithm.

The difference between the Delaunay triangulation algorithm and Poisson reconstruction algorithm is shown in Figure 2. Delaunay triangulation cannot guarantee the complete closure of the constructed mesh model, and the voids in the mesh model can cause volume calculation errors. Compared with the former, the latter is more complex, but the mesh model constructed by the Poisson reconstruction algorithm has water tightness and is more applicable to volume calculation.

The research on contactless calculation of livestock volume is in the exploratory stage. As the surface point cloud of livestock is complex and massive, the existing research mainly uses model reconstruction methods to calculate livestock volume. In 2016, Cheng proposed an improved Delaunay triangulation algorithm to construct a cattle point cloud mesh model and combined it with the tetrahedral summation method to calculate cattle volume [50], but this algorithm was not applied to real cattle point clouds and its accuracy was not verified. In 2022, Guo et al. [51] constructed a pig body mesh model through Delaunay triangulation and incorporated it with the triangular prism summation method to calculate the pig body volume. The body weight was estimated based on the volume and achieved good results in 20 test samples, with an estimated relative error within 8%.

At present, there are many methods for calculating the volume of irregular object point cloud models, but research on the volume computation of pig body point clouds is very limited. Moreover, the method of calculating the volume of livestock point clouds has high requirements in that the quality of the collected point clouds, the accuracy, and the robustness of volume calculation need to be further improved.

The present study intends to register and fuse pig body point clouds collected by three depth cameras that capture the data from different angles simultaneously. After preprocessing such as target point cloud extraction and denoising, an improved Poisson reconstruction algorithm is employed to construct a meshed 3D model of the pig body for volume calculation. Subsequently, a regression model is established based on the volume calculation results of the pig body point clouds to estimate the body weight.

## 2. Materials and Methods

### 2.1. Experimental Data Acquisition and Algorithmic Process

The pig point cloud data in this study were acquired from Jingbao Food Company, affiliated with Wens Foodstuff Group Co., Ltd., Heyuan City, China from July to August in 2022. A total of 479 sets of point cloud data were collected experimentally from 58 Landrace pigs. The target pigs were on an empty stomach for 2 h prior to the point cloud and weight data collection, and the pig weight ranged from 83 kg to 132 kg.

**Figure 3:**
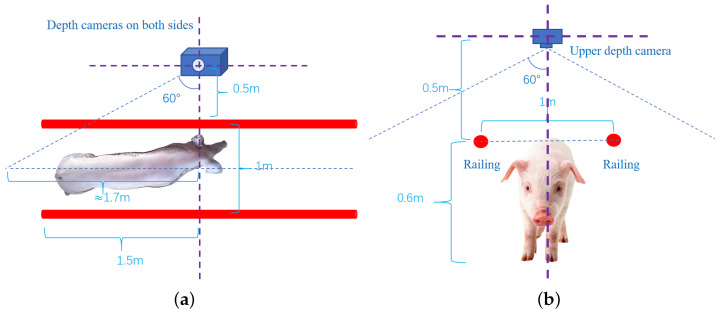
Schematic diagram of camera placement position. (**a**) Schematic diagram of depth cameras fixed on both sides. (**b**) Schematic diagram of the depth camera fixed from above.

**Figure 4:**
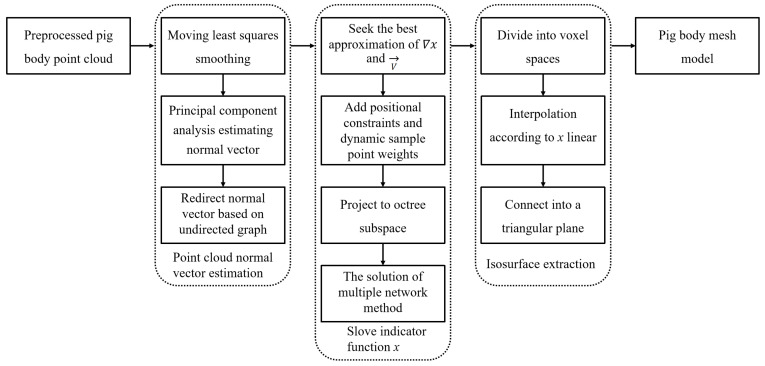
Flowchart of improved Poisson reconstruction algorithm.

Three depth cameras collect local point clouds of pig bodies from different angles (shown in Figure 3), with each local point cloud located in different coordinate systems. Therefore, it is necessary to use 3D point cloud registration technology to unify the three local point clouds into the same world coordinate system and fuse them into one complete pig point cloud [27]. The point cloud also includes the railings, the ground in the data acquisition area, as well as various noise points and outliers following registration and fusion. Consequently, first extracting the target pig body point cloud and then denoising it are required, and for the specific operation, refer to previous research conducted by [52]. After obtaining high-quality point cloud data of pig bodies, an improved Poisson reconstruction algorithm is employed to process these point cloud data by constructing a mesh-based three-dimensional model of the pig body. Subsequently, this model is decomposed into multiple tetrahedra to facilitate the calculation of the pig body volume. The algorithmic process of this study is illustrated in Figure 4.

### 2.2. Validation of Pig Body Volume Calculation Method

**Figure 5:**
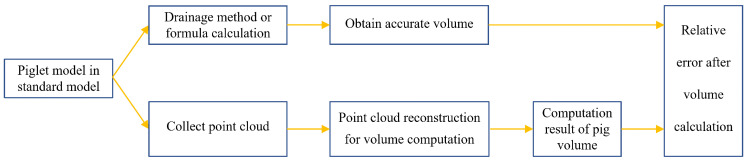
Testing the accuracy of the volume calculation method applied to the standard point cloud model and piglet model.

**Figure 6:**
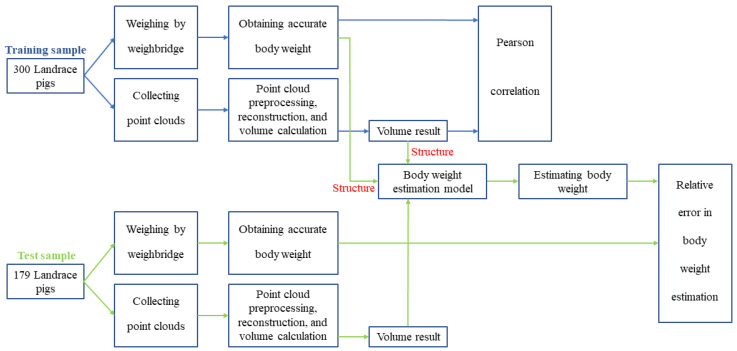
Volume calculation and body weight estimation process for experimental pig point cloud dataset (the blue line depicts the process of obtaining the Pearson correlation coefficient, whereas the green line illustrates the process of calculating the relative error in the estimated body weight).

All experiments were conducted in conformity with the guidelines on animal research established by the laboratory Animal Ethics Committee of South China Agricultural University (reference number: 2020G008). Due to the difficulty of obtaining corresponding volume data from real-time collected pig body point clouds, we verified the feasibility and accuracy of the pig body volume calculation method proposed in this study and focused on three aspects:

(1) The cube, cylinder, the Stanford rabbit [49] standard model, and the piglet model with known accurate volume values were used as test samples for the accuracy of our volume calculation method. The application of the standard point cloud model and piglet model in our experiment is shown in Figure 5.

(2) The correlation between the volume of real pig point cloud and body weight was calculated. By randomly selecting 300 test samples of pig body point clouds collected in the experiment, the volume calculation method in this article was utilized to acquire the volume of the target pig body point cloud, and the Pearson correlation coefficient between pig body volume and pig body weight was analyzed to confirm the strong correlation between body weight and the volume of the pig body point cloud, as shown in Figure 6.

(3) A linear regression weight estimation model was established based on the relationship between the volume calculation results and body weight in the sample set with the point cloud of 300 pigs, as shown in Figure 6. Subsequently, 179 sets of pig body point clouds were used as test samples to verify the accuracy of the volume calculation results using the relative error of body weight estimation.

### 2.3. Pig Point Cloud Smoothing

**Figure 7:**
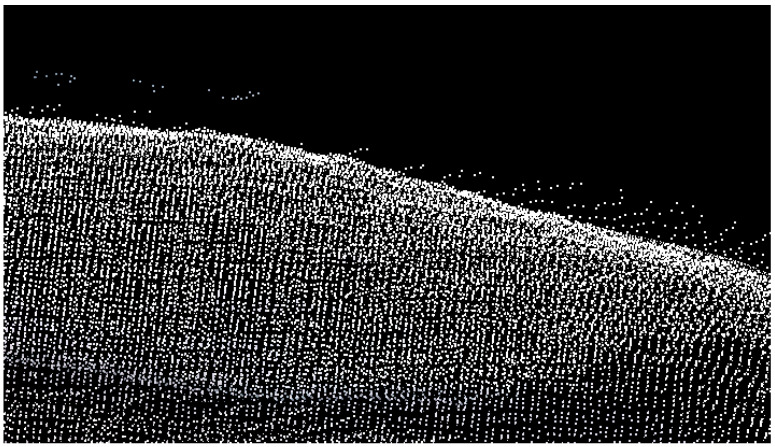
Burr noise points on the surface point cloud of pig body without smoothing treatment.

After pre-treatment, there is still a small portion of burr noise on the surface of the pig body point cloud, as shown in Figure 7. These burrs and noise points can cause sharp areas on the surface of the pig point cloud, resulting in deviations in subsequent point cloud normal vector estimation and distortion of the mesh model reconstructed by the Poisson algorithm.

To solve the above problems, we utilize the moving least squares method within a local range to fit the point cloud on the pig body surface into a smooth curved surface. The fitting function $u_{0}\left(x\right)$ for a point $x_{0}$ in the point cloud can be defined as [53]

(1) $$u_{0}\left(x\right)=\sum_{j=1}^{m}a_{j}\left(x_{0}\right)\times p_{j}\left(x\right)$$

Among them, $a_{j}$ is a set of coefficients for the fitting function, and $p_{j}\left(x\right)$ is a set of basis functions. Generally speaking, the quadratic basis function in three-dimensional space can use $p^{T}$ as follows:

(2) $$p^{T}=\left(1,x,y,z,xy,zx,yz,x^{2},y^{2},z^{2}\right)$$

To minimize the weighted sum of squares difference between the values of the sampling points $x_{0}$ and the values of the fitting function neighboring the sampling points, an optimization model *J* is established

(3) $$J=\sum_{b=1}^{k}w\left(x_{0}-x_{b}\right){\left[u_{0}\left(x_{0}\right)-u_{b}\right]}^{2}$$

where *k* is the number of neighboring sampling points of point $x_{0}$, $x_{b}$ is a neighboring sampling point of point $x_{0}$, $u_{b}$ is the value of the neighboring sampling point at $u(x_{0})$, and $w(x_{0}-x_{b})$ is the distance weight function. When point $x_{b}$ is closer to point $x_{0}$, the weight value w(x) increases, and vice versa.

**Figure 8:**
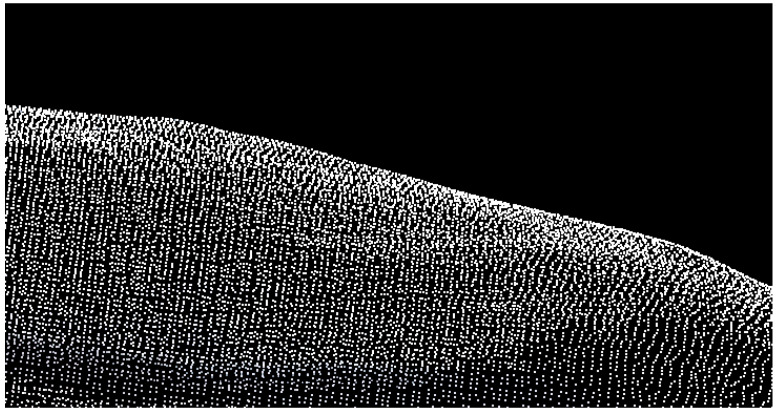
Smoothed point cloud on pig body surface.

When the derivative value of model *J* over a(x) is 0, the fitting function $u(x_{0})$ can be obtained. Based on $u(x_{0})$, the fitting function and its value can be calculated at any point to acquire a smoothed point cloud on the pig body surface, as shown in Figure 8.

### 2.4. Estimating Normal Vector via Principal Component Analysis

Before reconstructing the three-dimensional model of the pig body, the normal vector of the pig body point cloud is first calculated using principal component analysis. We select a point $x_{0}$ in the point cloud to estimate its normal vector, and a local region with *k* nearest points of $x_{0}$ is formed. When the region is small and smooth enough, it can be regarded as a plane, and the normal vector of point $x_{0}$ is the normal vector of that plane. We can transform finding a plane normal vector into finding a normal vector so that the projection points from points in the domain where the normal vector belongs to the vector are the most concentrated, that is, the projection variance of all points in a certain region in the vector direction is the smallest. Assisted by principal component analysis for constructing the minimum objective function and obtaining the normal vector, the following Equation (4) is used:

(4) $$\min_{∥n∥=1}\sum_{i=1}^{k}{\left(\left(x_{i}-x_{0}\right)n\right)}^{2}$$

The process of solving Formula (4) can be transformed into eigenvalue decomposition of the covariance matrix *C* composed of local domain point sets:

(5) $$C=\frac{1}{k}X^{T}X$$

Among them,

(6) $$X\;=\left(\begin{array}{c} \left(x_{1.x}-x_{0.x}\right)\left(x_{1.y}-x_{0.y}\right)\left(x_{1.z}-x_{0.z}\right)\\ \vdots \\ \left(x_{k.x}-x_{0.x}\right)\left(x_{k.y}-x_{0.y}\right)\left(x_{k.z}-x_{0.z}\right) \end{array}\right)$$

The minimization objective function (4) can be rephrased as [54]:

(7) $$\underset{∥n∥=1}{min}n^{T}Cn$$

The eigenvector corresponding to the minimum eigenvalue of the covariance matrix *C* represents that the variance is the smallest in this direction, and the projection of regional points is the most intensive, namely, the normal vector estimation value of the sampling point $x_{0}$.

### 2.5. Redirected Point Cloud Normal Vector

Principal component analysis is a local fitting method that can only estimate the straight line where the normal vector of a point cloud is located, but this algorithm cannot distinguish whether the direction of the normal vector is inside or outside the point cloud, nor can it acquire the correct orientation. Therefore, the point cloud normal vector estimated by principal component analysis will lead to opposite orientations between adjacent normal vectors. From a global perspective, provided that the normal vector direction of the point cloud faces partly inward and partly outward, errors are likely to occur in subsequent point cloud reconstruction.

First, this study stipulates that the normal vector towards the interior of the point cloud is the correct orientation, while the normal vector towards the exterior of the point cloud is the wrong orientation. The classic point cloud normal vector redirection is constructing an undirected graph based on neighborhood distance, propagating the point cloud normal vector as the correct direction. The method follows this principle: assuming that two points are close enough, they can be considered to be points on the same plane, and their normal vectors should have approximately identical orientation. We construct an undirected graph in a point cloud, with each edge having a weight value of $E_{i,j}$:

(8) $$E_{i,j}\;=\;\psi \left(i,j\right)•\omega \left(x_{i}-x_{j}\right)$$

where $\omega \left(x_{i}-x_{j}\right)$ is the distance weight function; ψ(i,j) is the correlation between the normal vector of point *i* and the normal vector of point *j*; $\psi \left(i,j\right)=<\vec{n_{i}},\vec{n_{j}}>$ represents the inner products of $\vec{n_{i}}$; and $\vec{n_{j}}$. The more consistent the orientation of the normal vectors of point *i* and point *j* is, the larger ψ(i,j). We select a vertex of a dimension in the point cloud as the starting point, specifying that the normal vector orientation of the vertex is consistent with the negative direction of the dimension. Then we propagate the normal vector orientation in the direction of generating the maximum spanning tree. The problem of redirecting point cloud normal vector is transformed into the problem of optimizing the maximum spanning tree with a weighted undirected graph.

**Figure 9:**
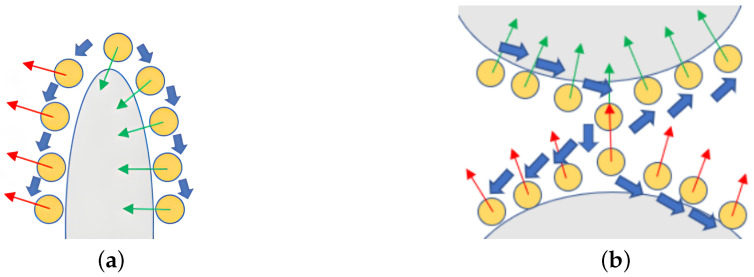
Construction of undirected graph based on neighborhood distance (specific example: erroneous propagation of normal vector, with green arrows indicating correct orientation and red arrows indicating incorrect orientation). (**a**) Sharp points in point clouds. (**b**) Thin planes in point clouds.

However, the method of constructing an undirected graph based on neighborhood distance is only applicable to ideal smooth point clouds. As shown in Figure 9, incorrect normal vector orientations may occur at sharp points and on thin planes. Due to the constraints of the distance weight function, the incorrect normal vector orientations are prone to propagate to neighboring points. To address the above issues, Jakob et al. [55] proposed constructing a KNN graph formed by connecting *K* nearest neighbor points of all points to redirect the point cloud normal vector.

Assuming that two adjacent points are connected as an edge, then this edge should act approximately as a tangent line to the plane on which it is located, perpendicular to the normal vector. Therefore, it is necessary to provide new constraints for the weight function $E_{i,j}$ of the edge, and the modifications to $\psi \left(i,j\right)$ are as follows:

(9) $$\psi \left(i,j\right)=<{\vec{n_{i}}}^{\prime },\vec{n_{j}}>,$$

(10) $${\vec{n_{i}}}^{\prime }=\vec{n_{i}}-\frac{p_{i}-p_{j}}{∥p_{i}-p_{j}∥}<\frac{p_{i}-p_{j}}{∥p_{i}-p_{j}∥},\vec{n_{i}}>$$

**Figure 10:**
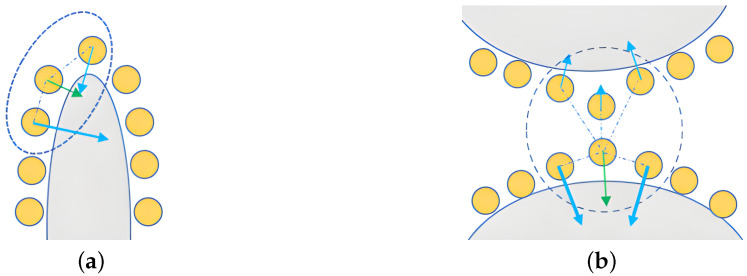
Constructing KNN graph to redirect point cloud normal vector (the blue arrows represent the known normals, while the green ones indicate the normals of the points to be solved; the longer the normal vector, the greater the weight). (**a**) Sharp points in point clouds. (**b**) Thin planes in point clouds.

When the vector formed by point *i* and point *j* and the normal vector $\vec{n_{i}}$ of point *i* are closer to perpendicular, the values of $\psi \left(i,j\right)$ become larger. As shown in Figure 10, a KNN graph is constructed to redirect the point cloud normal vector at the sharp points and on the thin planes of the point cloud. When the angle between the line (formed by neighboring points and the redirected points) and the normal vector of the neighboring point is smaller, it indicates that the normal vector orientation of the neighboring point is less trustworthy, and therefore, the obtained weight value is smaller. However, when the line is close to perpendicular to the normal vector, it signifies that the normal vector orientation of the neighboring point is more trustworthy, and therefore, the obtained weight value is larger. Compared with traditional methods, the new constraint method is more robust in sharp points and on thin planes of point clouds.

### 2.6. Reconstructing Mesh Model

#### 2.6.1. Surface Reconstruction Based on Poisson Equation

**Figure 11:**
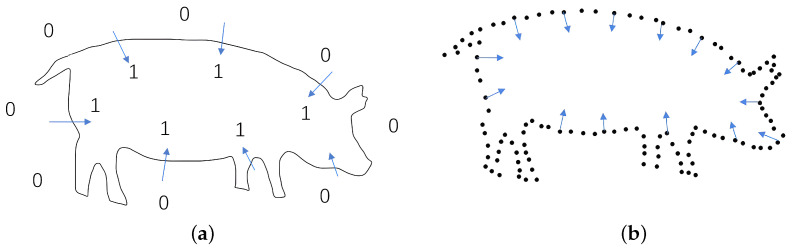
Relation between normal vector field of pig point cloud and gradient of exponential function. (**a**) Gradient field of indicator function χ. (**b**) Normal vector field of pig point cloud.

Poisson reconstruction can reconstruct the curved surface of a point cloud by constructing the indicator function, and the specific idea is seeking the best approximation in the gradient field of the indicator function to the point cloud vector field. The value of the indicator function χ in the inner space of the curved point cloud surface is 1, and the value of the function in the outer space of the point cloud surface is 0, as shown in Figure 11. Obviously, the gradient of the indicator function χ only has non-zero value on the curved point cloud surface, so solving the point cloud surface function can be converted into solving the indicator function χ.

We specify a normal vector field $\vec{V}$ to solve the indicator function χ, so that the gradient field ∇χ of indicator function χ is infinitely close to the normal vector field $\vec{V}$, that is, $\nabla \chi -\vec{V}$ is infinitely close to 0. Therefore, there is a minimization function E(χ):

(11) $$E\left(\chi \right)=min\;\|\nabla \chi -\vec{V}\|$$

The theorem mentioned above states that the indicator function χ has a discontinuity from 0 to 1 in the orthogonal direction of the curved point cloud surface, resulting in an infinite value when its gradient field is calculated. To overcome this issue, it is necessary to use a smoothing filter function. According to the divergence theorem, the gradient field of the smoothed indicator function is equal to the normal vector field of the smoothed surface.

During the solution process, as point cloud data are discrete in three-dimensional space, we need to obtain the normal vector field $\vec{V}$ through discrete approximation. After solving for $\vec{V}$, $\nabla \chi =\vec{V}$ is not directly soluble because $\vec{V}$ is not necessarily an irrotational field and cannot be integrated directly. Therefore, the problem is reformulated as a least-squares minimization problem to find the best solution:

(12) $$E\left(\chi \right)=\int {∥\nabla \chi \left(p\right)-\vec{V}\left(p\right)∥}^{2}dp$$

When E(χ) attains its minimum value, the Poisson equation is satisfied.

(13) $$\Delta \chi =\nabla •\vec{V}$$

Among them, Δ represents the Laplace operator, and ∇• represents the divergence operator.

#### 2.6.2. Octree Space Discretization

**Figure 12:**
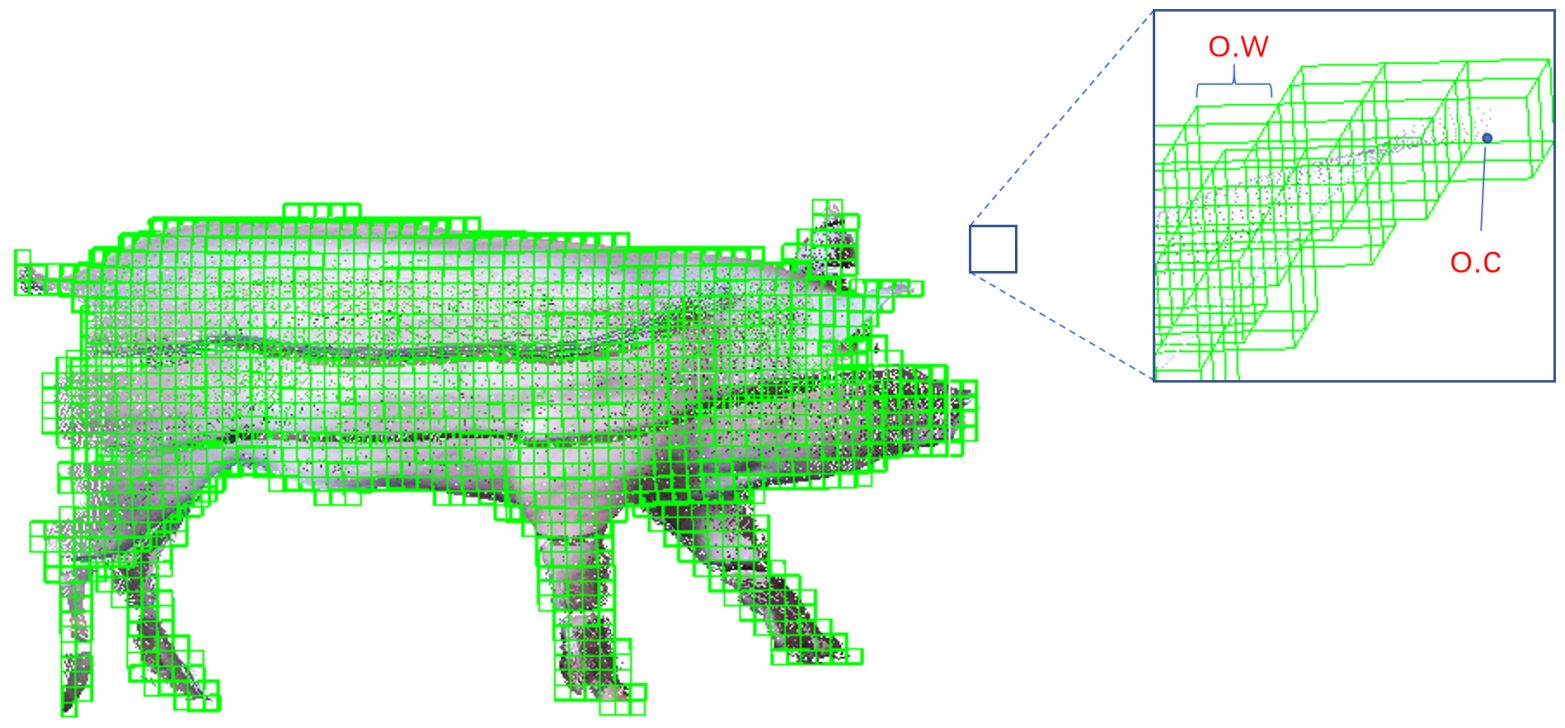
Octree space division of pig point cloud.

Solving Poisson’s equation requires discretization of the Poisson problem. This paper uses octree to partition the entire function space. Let the octree be *O*; for every node *o* on the octree, o∈O, there is a node function $F_{o}$, and the node function is composed of a base function *F*. It is required that the normal field $\vec{V}$ of the point cloud and the indicator function χ can be expressed by the linear combination of the node function $F_{o}$. Then, solving the indicator function χ can be transformed into solving the linear combination of the $F_{o}$. As shown in Figure 12, assuming the depth of the octree is *D*, the center of node *o* is o.c, and the node width is o.w, then the node function *o* can be defined as:

(14) $${\;F}_{o}\left(q\right)=\;F\left(\frac{q-o.c}{o.w}\right)\frac{1}{o.w^{3}}$$

The basis function *F* is actually a smoothing function centered around the origin, which can be defined as

(15) $$F\left(q\right)=\tilde{F}\left(\frac{q}{2^{D}}\right)$$

To further improve the operational efficiency of the algorithm, *F* will be defined as follows:

(16) $$F\left(x,y,z\right)\equiv {\left(B(x)B(y)B(z)\right)}^{*n}$$

Among them, the B-spline basis function B(ω) has a constant value of 1 only when ω is in the interval (−0.5, 0.5), and the other intervals are 0. *n represents n-fold convolution, and when *n* approaches infinity, F(q) approximates the Gaussian function infinitely.

The normal field $\vec{V}$ can be computed by using the normal vectors of the neighboring points. Subsequently, the indicator function χ can be obtained by solving the Poisson equation in reverse. However, although both $\vec{V}$ and χ can be defined by basis functions in the function space of an octree, Δχ and $\nabla •\vec{V}$ may not necessarily be in the same function space. Therefore, it is also imperative to minimize the distance between the projection of Δχ and $\nabla •\vec{V}$ in the octree function space:

(17) $$\sum_{o\epsilon O}{∥\langle \Delta \chi -\nabla •\vec{V},F_{o}\rangle ∥}^{2}=\sum_{o\epsilon O}{∥\langle \Delta \chi ,F_{o}\rangle -\langle \nabla •\vec{V},F_{o}\rangle ∥}^{2}$$

Let *v* be an |o|-dimensional vector and the coordinate value $v_{0}$ of the o-th node be $\langle \nabla •\vec{V},F_{o}\rangle$. The solution of the indicator function χ can be solved by approximating *v* with the Laplace operator for projection of χ in function space and the vector composed of $F_{0}$ [56].

#### 2.6.3. Isosurface Extraction

In Poisson reconstruction, to make the isosurface more approximate to the point cloud set, the average estimated value of the indicator function is used as the threshold $\mu_{iso}$ of the isosurface:

(18) $$\mu_{iso}=\frac{1}{\left|S\right|}\sum_{s\in S}\chi \left(s.p\right)$$

#### 2.6.4. Improved Poisson Equation Surface Reconstruction

The Poisson reconstruction algorithm directly seeks the best approximation between the gradient field and the normal field of the indicator function and uses a global offset to correct the error, which will lead to errors between the curved point cloud surface constructed by the Poisson reconstruction algorithm and the real object surface. The accuracy of estimating the normal vector of a point cloud depends on the quality of the point cloud, but the normal field is not consistently reliable. Therefore, this article considers adding position constraints to correct the position of the reconstructed point cloud surface. The improved Poisson reconstruction algorithm adds position constraints based on Poisson reconstruction, and its definition of a new minimization function E(χ) consists of gradient constraints and position constraints:

(19) $$E\left(\chi \right)=\int {∥\nabla \chi \left(p\right)-\vec{V}\left(p\right)∥}^{2}dp+\lambda \frac{Area(S)}{\sum_{p\in S}\tau (p)}\sum_{p\in S}\tau (p)\chi^{2}\left(p\right)$$

where λ is the weight factor for balancing gradient constraints and position constraints, *S* is the point cloud sample, the point cloud vector field is $\vec{V}$, Area(S) is the reconstruction area near the input sample point, and τ(p) is the sample point weight. This article adopts dynamic sample point weights and dynamically adjusts the sample point weights based on the credibility of the normal vector. Assuming there are *n* adjacent points in the neighborhood of point *p*, the sample point weight τ(p) is defined as

(20) $$\tau \left(p\right)=\frac{\sum_{i=0}^{n}g(cos\theta )}{n}$$

Among them, when g(x) is in x∈[0,1], $g\left(x\right)=cos\theta =\frac{\vec{Np}\times \vec{Ni}}{\left|\vec{Np}\right|\left|\vec{Ni}\right|}$, where θ is the angle between $\vec{Np}$ and $\vec{Ni}$, when g(x) is in x∈[−1,0], g(x) = 0. When the angle between the normal vector of point *p* and the normal vector of neighboring points is smaller, it can be considered that the accuracy of the normal vector of point *p* is higher, and the value of weight τ(p) is closer to 1. On the contrary, provided that the angle between the normal vector of point *p* and the normal vector of neighboring points is closer to a right angle, it is considered that the normal vector of point *p* is more untrustworthy, and the value of weight τ(p) is closer to 0.

Let ${\langle .,.\rangle }_{{\left[0,1\right]}^{3}}$ represent the standard inner product on the function space of the octree, such as

(21) $${\langle F,G\rangle }_{{\left[0,1\right]}^{3}}=\int F(p)-G(p)dp$$

(22) $${\langle \vec{U},\vec{V}\rangle }_{{\left[0,1\right]}^{3}}=\int \vec{U}\left(p\right)-\vec{V}\left(p\right)dp$$

and let ${\langle .,.\rangle }_{\left(\tau ,S\right)}$ represent the weighted sum of function values in the function space of the octree, which has bilinear, symmetric, non-negative, and semi-stereotyped forms, such as

(23) $${\langle F,G\rangle }_{\left(\tau ,S\right)}=\frac{Area(S)}{\sum_{p\in S}\tau (p)}\sum_{p\in S}\tau (p)F\left(p\right)G\left(p\right)$$

Thus, Equation (19) can be simplified into the following expression:

(24) $$E\left(\chi \right)={\langle \vec{V}-\nabla \chi ,\vec{V}-\nabla \chi \rangle }_{{\left[0,1\right]}^{3}}+\lambda {\langle \chi ,\chi \rangle }_{\left(\tau ,S\right)}$$

Furthermore, Formula (24) can also be expressed in the form of Poisson’s equation:

(25) (Δ−λI)χ=∇•V

Among them, *I* is a positive definite operator used to convert the different space of the gradient constraint and position constraint where they are located.

### 2.7. Volume Calculation of Three-Dimensional Mesh Models

The tetrahedral summation method is a three-dimensional volume calculation method marked by wide applicability and high accuracy. A three-dimensional mesh model is a model composed of vertices and edges, with vertices connected to each other to form triangular facets; hence, it is also known as a triangular facet model. For each triangular facet in the network model, connecting three vertices and the origin forms a tetrahedron, which is the basic unit of the tetrahedron summation method.

**Figure 13:**
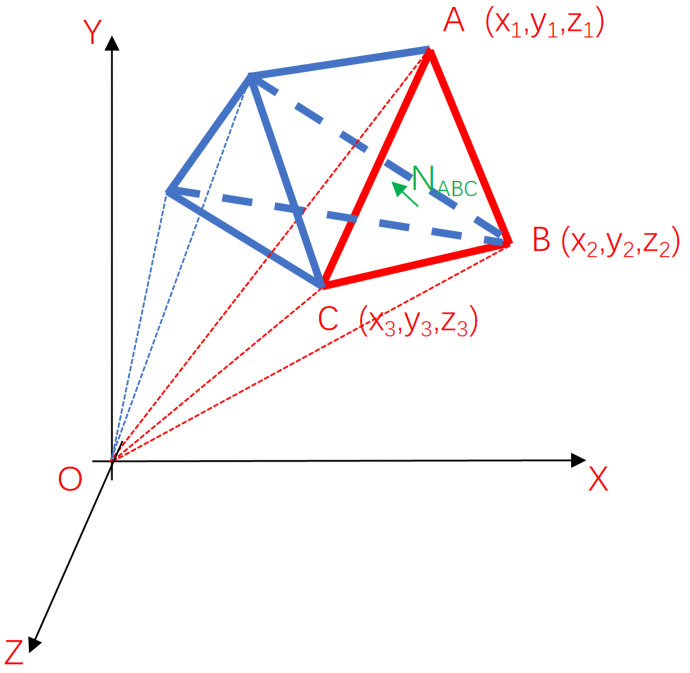
Calculation of tetrahedral OABC volume.

To calculate the volume of a tetrahedron, first the normal vector of the triangular facet is calculated, which can be obtained by the cross product of the vectors of two edges, as shown in Figure 13. For $\vec{N_{ABC}}$, the coordinates of vertices A, B, and C are $\left(x_{1},y_{1},z_{1}\right)$, $\left(x_{2},y_{2},z_{2}\right)$, and $\left(x_{3},y_{3},z_{3}\right)$, respectively. Then, the vector $\vec{AB}=\left(x_{2}-x_{1},y_{2}-\;y_{1},\;z_{2}-\;z_{1}\right)$, vector $\vec{BC}=\left(x_{3}-x_{2},y_{3}-\;y_{2},\;z_{3}-\;z_{2}\right)$, and normal vector $\vec{N_{ABC}}=\vec{AB}\times \vec{BC}$. In this article, the normal vector of the triangular facet is specified to face the interior of the tetrahedron. The volume of each tetrahedron is assigned a positive sign or a negative sign. The rule for assigning a positive sign or a negative sign is described as follows: when the normal vector of the triangular facet and the origin are on the same side, a positive sign is assigned. Conversely, a negative sign is assigned. Furthermore, the judgment basis can be the sign of the result derived from inner product of the vector pointing from vertex to origin and the normal vector of the triangular facet. As shown in Figure 13, the tetrahedral volume formed by the triangle facet ABC and the origin can be expressed as

(26) $$V_{OABC}=\frac{\vec{AO}*\vec{N_{ABC}}}{\left|\vec{AO}*\vec{N_{ABC}}\right|}*\frac{1}{6}\left(-x_{3}y_{2}\;z_{1}+x_{2}y_{3}z_{1}+x_{3}\;y_{1}\;z_{2}-x_{1}y_{3}\;z_{2}-x_{2}y_{1}z_{3}+x_{1}y_{2}z_{3}\right)$$

Furthermore, provided that *i* represents the *i*th triangular facet and *I* represents the first vertex of the triangular facet, the volume of the *i*th tetrahedron can be expressed as

(27) $$V_{i}=\frac{\vec{IO}*\vec{N_{i}}}{\left|\vec{IO}*\vec{N_{i}}\right|}*\frac{1}{6}\left(-x_{i3}y_{i2}\;z_{i1}+x_{i2}y_{i3}z_{i1}+x_{i3}\;y_{i1}\;z_{i2}-x_{i1}y_{i3}\;z_{i2}-x_{i2}y_{i1}z_{i3}+x_{i1}y_{i2}z_{i3}\right)$$

**Figure 14:**
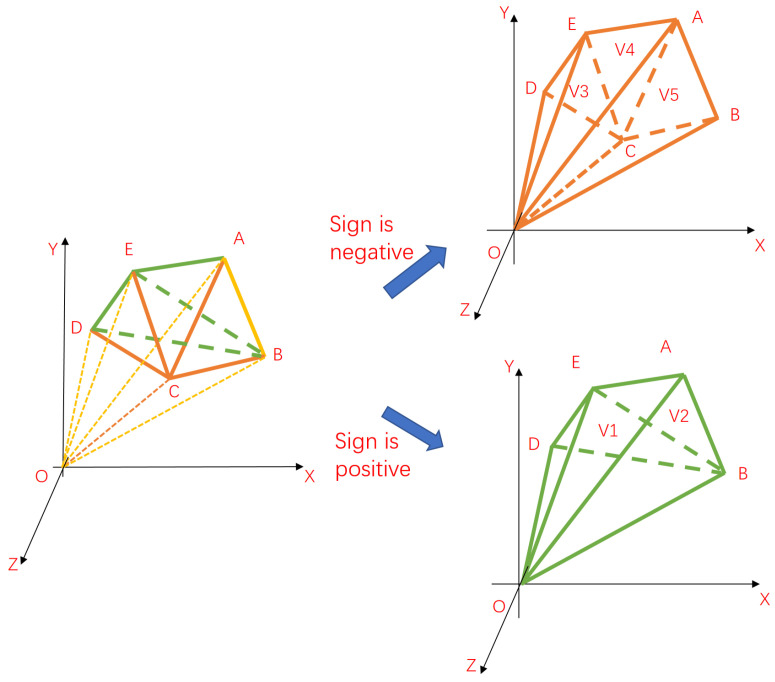
Tetrahedral summation method. (The pentahedron ABCED is decomposed into positive volume area and negative volume area, $V_{total}=V_{1}+V_{2}-V_{3}-V_{4}-V_{5})$.

As shown in Figure 14, the volume of an irregular network model can be decomposed into positive and negative volume regions in the tetrahedral summation method. By summing the volumes of all signed triangles, the volume of the network model can be obtained, and the result of the volume is unrelated to whether the origin is inside the model or not. Therefore, the volume of the network model can be expressed as

(28) $$V_{total}=\;\sum_{i}Vi$$

**Figure 15:**
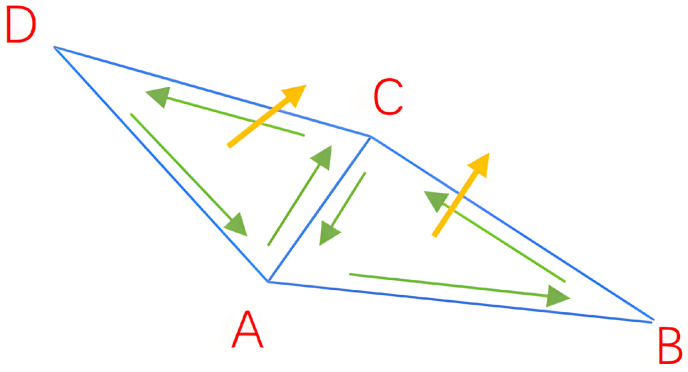
Judging the normal vector orientation according to the right-hand rule.

It is worth noting that calculating the three-dimensional volume involves computing the normal vector of the surface. Due to the ambiguity of the normal vector, it is essential to determine the direction of the normal vector. If the vertex numbers in the constructed network model data are in order, the orientation of the surface normal vector can be judged by the right-hand rule, as shown in Figure 15. However, not all the vertex numbers in the network model data are orderly. If the right-hand rule cannot be applied, the normal vector orientation of the triangle facet is determined according to the point cloud that has previously been processed for normal vector redirection. By searching for the nearest point cloud of these three vertices in the point cloud through the spatial coordinates of the three vertices of a triangular facet, the normal vector orientation of the triangular patch can be identified based on the orientation of the normal vector in the point cloud.

## 3. Experimental Results and Analysis

### 3.1. Analysis of Point Cloud Reconstruction Results

This article respectively compares the effects of Poisson reconstruction and improved Poisson reconstruction algorithms, as well as the outcomes of comparison without adding a smoothing process.

**Figure 16:**
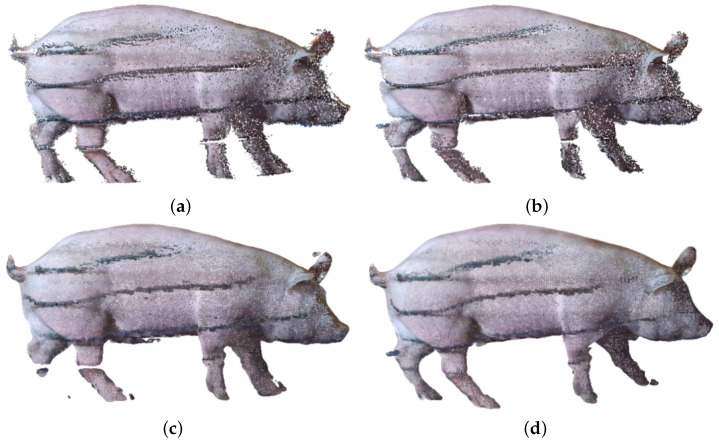

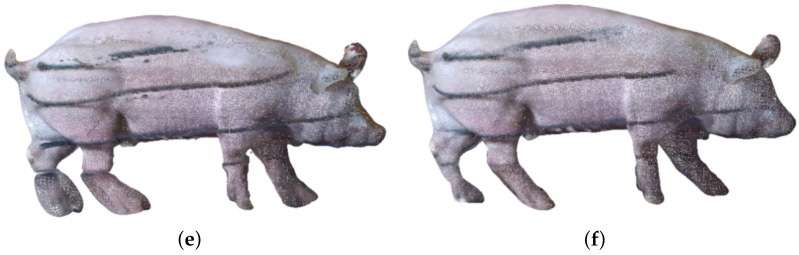
Comparison of different point cloud reconstruction methods. (**a**) Preprocessed pig body point cloud. (**b**) Smoothed pig body point cloud. (**c**) Traditional Poisson reconstruction. (**d**) Smoothing plus traditional Poisson reconstruction. (**e**) Improved Poisson reconstruction. (**f**) Smoothing plus improved Poisson reconstruction.

From the visualization results of point cloud reconstruction in Figure 16, it can be observed that there was a significant difference in the reconstruction effect between the smoothed and non-smoothed point clouds. As shown in Figure 16a,c,e, reconstruction errors may occur in the uneven surface area of the point cloud that had not been smoothed. For example, in the bottom area and the pig leg area of the pig body, the corresponding parts appear swollen or broken, which are erroneous phenomena during reconstruction. By contrast, the point cloud that had been smoothed, as shown in Figure 16b,d,f, had fewer errors in reconstruction. Our experimental results demonstrate that point cloud smoothing can indeed improve the accuracy of point cloud normal vector estimation, making point cloud reconstruction more effective.

Compared with Poisson reconstruction, the reconstruction effect of improved Poisson reconstruction is similar in the trunk part of the pig body, while in more complex areas on the point cloud surface, such as the pig leg area, the reconstruction effect differs significantly. The surface processed by Poisson reconstruction tends to contract inward on complex point cloud surfaces, as shown in Figure 16c,d, while improved Poisson can reconstruct surfaces with the outermost point cloud defined as the standard, as shown in Figure 16e,f.

**Figure 17:**
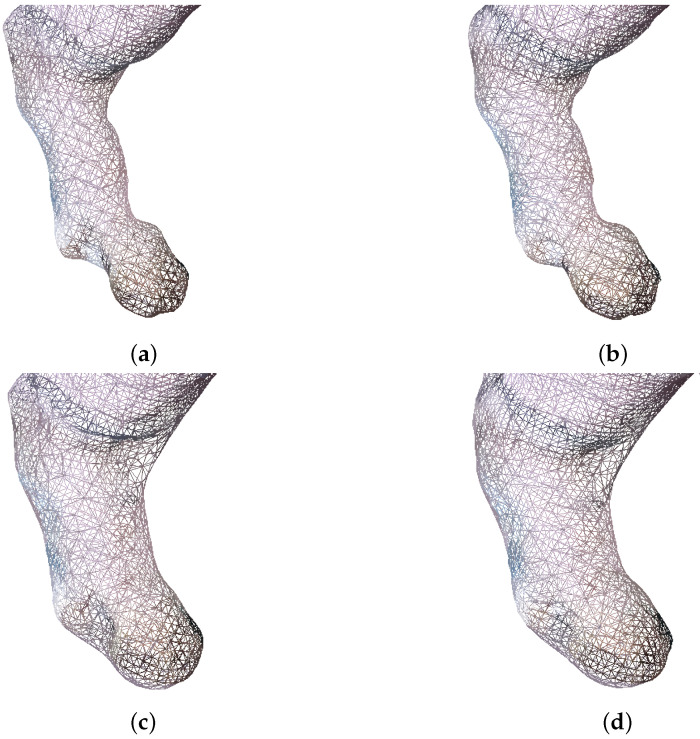
Comparison of pig leg area using different point cloud reconstruction methods. (**a**) Traditional Poisson reconstruction. (**b**) Smoothing plus traditional Poisson reconstruction. (**c**) Improved Poisson reconstruction. (**d**) Smoothing plus improved Poisson reconstruction.

The number of point clouds in the pig leg area is small; moreover, the pig leg point cloud may be partially missing due to being blocked by the railing in the data acquisition area. When the target pigs pass through the collection channel, the dust on the ground kicked up by their hooves causes many noise point clouds around the pig legs, constituting the most complex area in point cloud reconstruction and the area with the largest number of errors in volume calculation. The images of pig leg point clouds reconstructed by using different methods are shown in Figure 17. In Figure 17a,b, the pig legs reconstructed by Poisson contract inward, making the volume of the pig legs smaller compared to their actual volume. By comparison, the pig legs reconstructed by improved Poisson are fuller than those reconstructed by traditional Poisson and more in line with the actual pig legs, as shown in Figure 17c,d.

**Figure 18:**
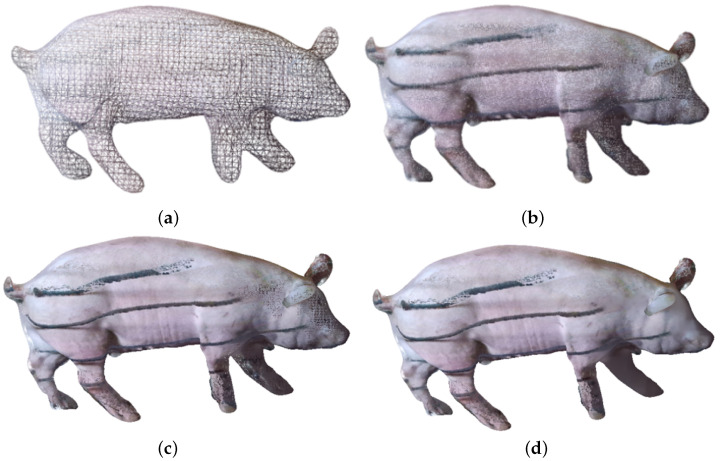
Comparison of improved Poisson reconstruction effects under different octree depths. (**a**) Octree depth, d = 6. (**b**) Octree depth, d = 8. (**c**) Octree depth, d = 10. (**d**) Octree depth, d = 12.

Prior to Poisson reconstruction, octree is used to perform discretization of voxels. The octree depth is an important parameter: the larger the octree depth parameter, the higher the resolution, and the richer the details. In Figure 18, the reconstruction results are visualized under different octree depths.

In improved Poisson reconstruction, when the depth of the octree is less than six, the reconstruction effect is significantly reduced, and the reconstructed pig body network structure is deformed, which means that when the depth of the octree is less than six, its resolution is not sufficient to reconstruct a network structure model of the pig body with good performance, as shown in Figure 18a. On the other hand, when the depth of the octree is greater than or equal to eight, the pig model reconstructed with the increase in the octree depth does not have obvious deformation. As a result, from a local perspective, the increase in the octree depth makes the reconstructed mesh model more refined and more detailed, with a smoother surface achieved.

As shown in Table 1, when the depth of the octree increased, the number of triangular facets of the mesh model increased significantly; so did the running time. At the same time, under the same octree depth, the number of triangular facets produced by the improved Poisson reconstruction was larger than that of the traditional Poisson reconstruction, which indicates that the surface details of the improved Poisson reconstruction are richer and the degree of reduction is higher than that of the traditional Poisson reconstruction. Based on the above experimental findings, the improved Poisson reconstruction algorithm with the octree depth set as eight is most suitable for reconstructing the pig mesh model.

### 3.2. Analysis of Volume Calculation Results

**Figure 19:**
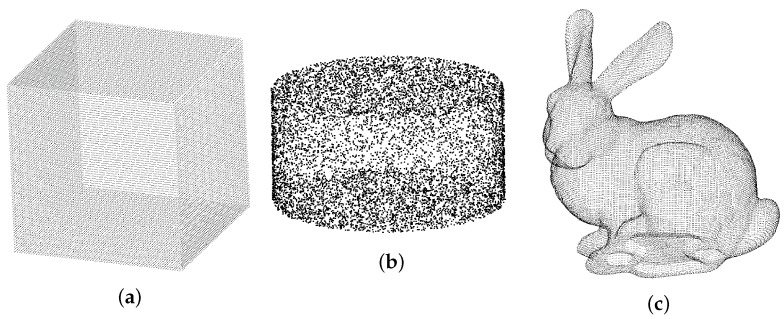
Standard models. (**a**) Cube. (**b**) Cylinder. (**c**) Rabbit.

In this article, the standard cube point cloud model, the standard cylinder point cloud model generated by the CloudCompare software (www.cloudcompare.org), and the classic Stanford rabbit [49] model (shown in Figure 19), which can calculate the true value of reliable volume, are used as references to test the accuracy of the volume calculation method proposed in this paper, and comparison is made between the slicing method of calculating three-dimensional volume and our method, with the slicing interval being the minimum spacing of point clouds.

Table 2 and Table 3 show that the slicing method had high computational accuracy when applied to calculating the volume of regular point cloud models, but there was a large error when calculating the volume of irregular and complex models. This can be attributed to the fact that the slicing integration method relies on the calculation of the slice contour; therefore, when handling complex models, a large error occurs in the calculation of the slice contour area. Additionally, the slice contour has internal and external contour problems as well as multiple contour problems [57], resulting in calculation accuracy errors. By contrast, constructing a mesh model to calculate volume had good computational accuracy in both regular and irregular models, signifying the feasibility and accuracy of using the tetrahedral summation method to calculate the volume of the mesh model. Further experiments showed that the increase in octree depth did not significantly improve the accuracy of volume calculation, but the increase in octree depth could simultaneously lead to a larger number of triangular facets and a greater possibility of over fitting.

**Figure 20:**
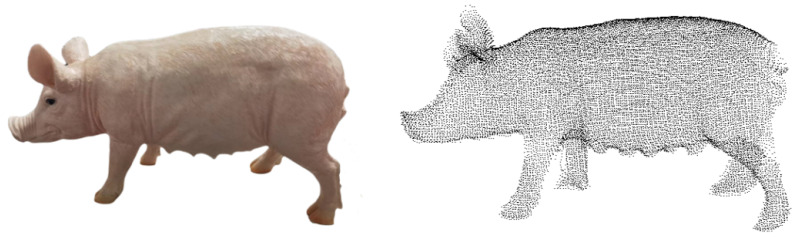
Piglet model.

Furthermore, to test the accuracy of the volume calculation method proposed in this article for irregular object volume calculation, a small pig body model with similar shape was used for testing experiments, as shown in Figure 20. First, the drainage method was adopted to calculate the true volume of the piglet model. Specifically, a transparent water bucket and a measuring tape were used. The first step is to immerse the piglet model completely in water; the second step is to calculate the volume of the discharged water to obtain its true volume. The average of ten measurement results is taken as the final result, and the true volume of the piglet model is 4537.3 cm^3^. In the third step, the point cloud reconstruction and volume calculation method proposed in this study is employed to calculate the volume of the piglet model.

According to Table 4, the volumes were obtained first by reconstructing mesh models via Poisson and improved Poisson, and then by using the tetrahedral summation method. The relative errors of the volume results were within 6%. The volume relative error obtained by improved Poisson reconstruction was 1% lower than that obtained by traditional Poisson reconstruction. The piglet model proves that the volume calculation method proposed in this paper has satisfactory accuracy, and the volume accuracy of the mesh model obtained by improved Poisson is higher than that of the mesh model obtained by traditional Poisson. Taking the improved Poisson reconstruction with an octree depth of eight as an example, under the condition of similar computation time (shown in Table 1), although its enhancement on the piglet model is only 1.5%, with increasing volume, a 1.5% error can lead to an increased variation in estimated weight. According to Equation (29), a 1.5% volume change may result in a weight variation of around 2 kg. Meanwhile, it can be found that when the octree depth is greater than six, the increase in octree depth has little impact on the final result of the volume calculation.

In order to verify the accuracy of the algorithm applied in real pig bodies, the strong correlation between animal real volume and real body weight data was utilized in this study [58], and the real body weight of pigs was accurately obtained by the weighbridge scale. This article establishes an estimation model based on the calculation results of pig body point cloud volume to estimate pig body weight, indirectly evaluating the error of pig body point cloud volume calculation by means of the deviation between the estimated weight value and the actual value.

**Figure 21:**
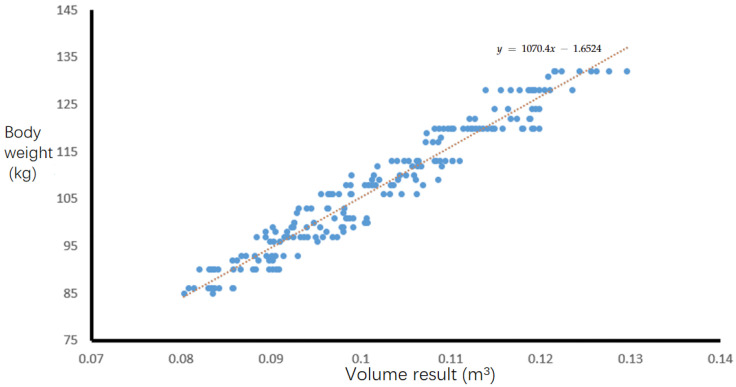
Scatter plot of point cloud volume calculation results and actual body weight of 300 pigs.

In the experiment, 479 pig point cloud sample data were divided into 300 training sample sets and 179 test sample sets. The training sample set was used to build the body weight prediction model, and the test sample was used to estimate the body weight. The improved Poisson reconstruction algorithm with an octree depth of eight was combined with the tetrahedron summation method to obtain the volume calculation results of 300 pigs, so as to draw the scatter plot of their volume and weight, as shown in Figure 21.

Figure 21 shows a linear relationship between the calculated volume of the pig point cloud and the actual body weight of the pig, with a Pearson correlation coefficient of r = 0.95, indicating a strong positive correlation between the calculation result of pig point cloud volume and the actual pig body weight. From these 300 training sample data, a linear regression prediction model is established. The linear regression equation can be expressed as f(x)=ax+b, where $a=\frac{\sum \left(x_{i}-\bar{x}\right)\left(y_{i}-\bar{y}\right)}{\sum {\left(x_{i}-\bar{x}\right)}^{2}}$, b=y−ax. Therefore, the mathematical relationship of the body weight estimation model is

(29) y=1070.4x−1.6524

Among them, $R^{2}$ represents the degree to which goodness of fit reflects linear fitting. The closer the goodness of fit is to 1, the higher the impact of *x* on *y*, and the better the fitting effect. The definition of $R^{2}$ is as follows:

(30) $$R^{2}\;=\;1-\frac{\sum {\left(y_{i}-f\right)}^{2}}{\sum {\left(y_{i}-\bar{y}\right)}^{2}}$$

According to Figure 21 and the goodness of fit $R^{2}$ = 0.921 of the linear regression equation, it can be concluded that the body weight estimation model has a good fitting effect on 300 training sample data. Using the same method proposed by us, the volume of 179 test sample sets was calculated, and the volume results were substituted into the body weight estimation model to estimate the body weight results, which were compared and analyzed with the corresponding weighbridge weighing.

**Figure 22:**
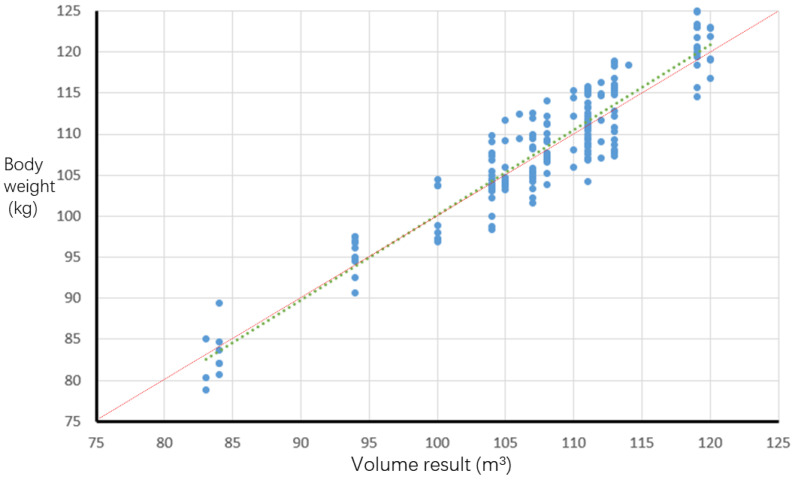
Scatter plot of estimated body weight and actual body weight of 179 pigs.

The scatter plot of the estimated body weight and actual body weight of pigs is shown in Figure 22. The blue dotted line represents the linear fitting straight line between the estimated body weight and the actual body weight of 179 pigs, while the red straight line represents the y=x function. The fitting straight line is very close to the y=x function, indicating that the estimation effect of the body weight estimation model is highly desirable.

**Figure 23:**
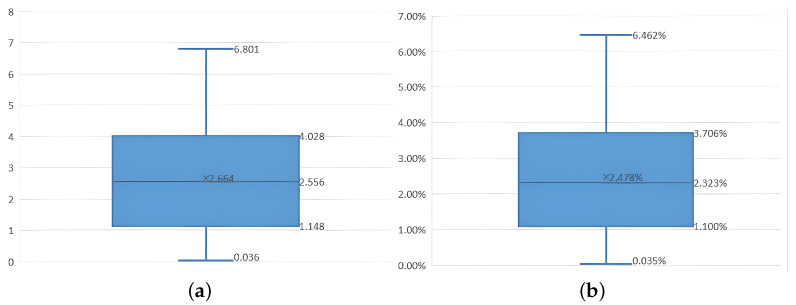
Box-plot of absolute error and relative error in body weight estimation model. (**a**) Absolute error of estimated body weight. (**b**) Relative error of estimated body weight.

Box-plots of absolute error and relative error for the body weight estimation model are shown in Figure 23. The maximum absolute error of body weight estimation was 6.801 kg; the minimum absolute error was 0.036 kg; the average absolute error was 2.664 kg. The upper quartile value of absolute error was 4.028 kg, while the lower quartile value was 1.148 kg. The maximum relative error of body weight estimation was 6.462%; the minimum relative error was 0.035%. The average relative error was 2.478%, and the upper quartile and lower quartile values of relative error were 3.706% and 1.100%, respectively. Through the above error analysis, it can be concluded that the body weight estimation model established based on the volume calculation results has superb estimation performance. Compared with the methods proposed in the existing literature, Guo et al. [51] used Delaunay triangulation to build a mesh model and then obtained the volume calculation result through the sum of three prisms. The average relative error of body weight estimated from the volume calculation results of 20 sample point clouds was 5.162%. Kongsro estimated the body weight of 71 pigs based on the projected area and the body height of pig body surface, and the root-mean-square deviation was 4.8% [15]. Compared with previous research results, the pig body volume calculation method proposed in this research has higher accuracy and further feasibility in predicting body weight.

## 4. Discussion

For large quantities of pig body point cloud data with complex surface contours, previous studies have typically focused on using 2D image-based or simple 3D reconstruction technology to obtain livestock volume data. However, limited by data dimensionality and complexity, these methods often fail to accurately capture the intricate features of the pig body surface. Additionally, the slicing method, one of the commonly used algorithms for point cloud volume calculation, is prone to potential errors as many factors are taken into consideration, such as inner fitting and outer contour fitting, multi-contour fitting, slicing direction, and slicing interval. Due to the missing parts in the pig body point cloud, pig body data acquisition is highly demanding. Moreover, the Monte Carlo method is not suitable for volume calculation of large animals like pigs.

In contrast, the method proposed in this paper calculates pig body volume by using an octree-enhanced Poisson model reconstruction, demonstrating the feasibility and accuracy of this approach through multiple sets of experiments. The specific work and main innovations of this paper are as follows:

(1) High-quality target pig point clouds are obtained by using the moving least squares method to further smooth pig body point cloud data for subsequent point cloud reconstruction and volume calculation.
(2) The maximum spanning tree is constructed based on the undirected graph to redirect pig body point cloud normal vector, thus improving the accuracy of the point cloud normal vector, which in turn makes our method conducive to reducing the erroneous reconstruction caused by the Poisson reconstruction algorithm in the complex area of the pig body point cloud.
(3) The improved Poisson reconstruction algorithm, with the addition of position constraints and dynamic sample point weights, can more accurately represent complex surfaces and more advantageously repair small-scale voids in the pig point cloud in actual scenes, and it can reduce the errors generated during reconstruction. Meanwhile, experimental outcomes show that superior reconstruction effects can be achieved in voids and the pig leg point cloud.
(4) By decomposing the reconstructed pig mesh model into multiple tetrahedrons, the signed volumes of all tetrahedrons are summed to obtain the mesh model volume. Through the experiments on the standard model, the relative errors of the volume calculation results were all less than 1%, which proves that the tetrahedron summation method has a high accuracy in calculating the volume of the standard model. According to the volume calculation experiment conducted on the piglet model after collecting its point cloud, the relative error of the volume result obtained from Poisson reconstruction was about 5.6%, while the relative error of the volume result obtained from improved Poisson reconstruction was about 4%. The 479 pig point cloud data sets collected from the pig farm were divided into 300 training samples and 179 test samples. An estimation model for body weight was first established by using the volume calculation results of the training sample point cloud. The pig body weight was subsequently estimated using the volume calculation results of 179 test samples. Finally, comparisons were made between the estimated pig body weight and the actual pig body weight for further analyses. The experimental results reveal that the maximum absolute error of body weight estimation was 6.801 kg; the average absolute error was 2.664 kg; the maximum relative error and the average value were 6.462% and 2.478%, respectively. Given the difficulty in obtaining live pig body volume, we put forward in this paper a method to indirectly verify the accuracy of pig body point cloud volume calculation, confirming that the correlation coefficient between pig body volume and pig body weight was 0.95.

In the past, obtaining livestock body volume data either manually or automatically has been a challenging research topic. As a new phenotype, the volume data can be used not only to estimate body weight more accurately, but also to evaluate meat density efficiently. Through point cloud segmentation, it can also be applied to the volume size prediction and proportion estimation of various body parts, which has new reference value in breeding management and selective breeding.

## 5. Conclusions

The present study presents an innovative approach to accurately reconstructing and calculating the volume of pig body point clouds, leveraging an improved Poisson reconstruction algorithm integrated with octree. Through a series of experiments and validations, we have demonstrated the feasibility and accuracy of our method, achieving significant advancements in livestock body volume estimation. By incorporating techniques such as moving least squares smoothing, normal vector adjustment, and tetrahedron summation for volume calculation, we have addressed challenges associated with irregular point cloud shapes and missing data, achieving impressive results with low relative errors.

Furthermore, the strong correlation between pig body volume and weight, as evidenced by a correlation coefficient of 0.95, underscores the utility of volume data in accurate weight estimation and efficient breeding management. This novel phenotype not only enhances weight estimation accuracy but also offers potential applications in meat density evaluation and body part proportion estimation through point cloud segmentation. Our research opens new avenues for precise livestock management and selective breeding strategies. In future studies, we aim to explore further advancements, particularly in segmenting various parts of the pig body point cloud for more comprehensive volume analysis.

## Figures and Tables

**Table 1:** Influence of octree depth on reconstruction time and number of facets.

Poisson Reconstruction			Improved Poisson Reconstruction	
Depth	Time (s)	Number of Triangular Facets	Time (s)	Number of Triangular Facets
6	0.328	8802	0.554	9322
8	2.251	109,350	2.343	122,734
10	7.854	451,246	8.192	458,122
12	12.891	493,294	16.408	546,662

**Table 2:** Volume calculation results of standard models.

Model	True Volume Value	Slicing Method	Poisson Reconstruction, d = 8	Poisson Reconstruction, d = 12	Improved Poisson Reconstruction, d = 8	Improved Poisson Reconstruction, d = 12
Cube	1	0.9999	1.0009	1.0009	0.9992	0.9992
Cylinder	3.1415	3.1483	3.126	3.126	3.1245	3.1244
Rabbit	753,955	936,250	756,517	756,501	755,526	755,515

**Table 3:** Relative error of volume calculation results of standard models.

Model	Slicing Method	Poisson Reconstruction, d = 8	Poisson Reconstruction, d = 12	Improved Poisson Reconstruction, d = 8	Improved Poisson Reconstruction, d = 12
Cube	0.01%	0.09%	0.09%	0.08%	0.08%
Cylinder	0.2164%	0.4933%	0.4933%	0.5411%	0.5443%
Rabbit	24.1784%	0.3398%	0.3376%	0.2083%	0.2069%

**Table 4:** Volume calculation results and errors of the piglet model.

Reconstruction Method	Volume Result	Absolute Error	Relative Error
Poisson reconstruction, d = 6	4788.3	251	5.532%
Poisson reconstruction, d = 8	4283.5	253.8	5.5936%
Poisson reconstruction, d = 12	4283.6	253.7	5.591%
Improved Poisson reconstruction, d = 6	4803.1	265.8	5.858%
Improved Poisson reconstruction, d = 8	4350.9	186.4	4.1081%
Improved Poisson reconstruction, d = 12	4352.1	185.2	4.0817%

## Data Availability

The data presented in this study are available on request from the corresponding author. The data are not publicly available due to ethical reasons.

## Abbreviations

**Specialized Terminology**	**Description**
Cutting	It refers to the process of dividing a large point cloud dataset into multiple subsets, which typically represent different objects or different parts of objects within the point cloud.
Cutting direction	When performing segmentation of three-dimensional point cloud data, a directional segmentation strategy can be adopted, which involves selecting one or more predefined directions for segmentation. These directions can align with the standard coordinate axes (X, Y, Z axes) of the point cloud data, or they can be non-axial directions selected based on specific scene requirements.
Cutting interval	It refers to the distance selected for segmentation along a specific direction in three-dimensional point cloud data.
Point cloud contour fitting	It refers to fitting a set of scattered three-dimensional point clouds into contours with specific geometric shapes using mathematical models.
Point cloud multi-contour	Contours of multiple independent objects appear simultaneously in a single point cloud dataset. In a complex scene’s point cloud, there may be multiple different objects, each with its own contours and boundaries.
Point cloud multi-contour fitting	It refers to the process of analyzing and processing complex point cloud data to identify and fit the multiple contours of these objects.
Slice	Point cloud slicing refers to subsets obtained by cutting three-dimensional point cloud data along specific directions, which can be viewed as polygonal prisms.
Slice contour	Slice contour defines the bottom contour of the prism.
Slice integration	It refers to a technique used to calculate the volume of three-dimensional point clouds. For more details, refer to Zhi et al. [36].
Slice interval, Slice spacing	The slice interval defines the height of the prism.
Slice projection	It refers to the process of projecting a prism onto a two-dimensional plane.
